# Editorial: Reviews in cardiac rehabilitation

**DOI:** 10.3389/fcvm.2024.1371750

**Published:** 2024-05-13

**Authors:** Melissa Tracy, Costantino Mancusi, Andrea Salzano

**Affiliations:** ^1^ Rush University Medical Center, Chicago, IL, United States; ^2^ Department of Advanced Biomedical Science, Federico II University Hospital, Naples, Italy; ^3^ Cardiology Unit, AORN A Cardarelli, Naples, Italy; ^4^ Cardiology, Glenfield Hospital, University Hospital of Leicester, Leicester, United Kingdom

**Keywords:** cardiac rehabilitation (CR), prevention, global issues, cardiovascular diseases, alternate options for cardiac rehabilitation

**Editorial on the Research Topic** Reviews in cardiac rehabilitation

**Figure 1:**
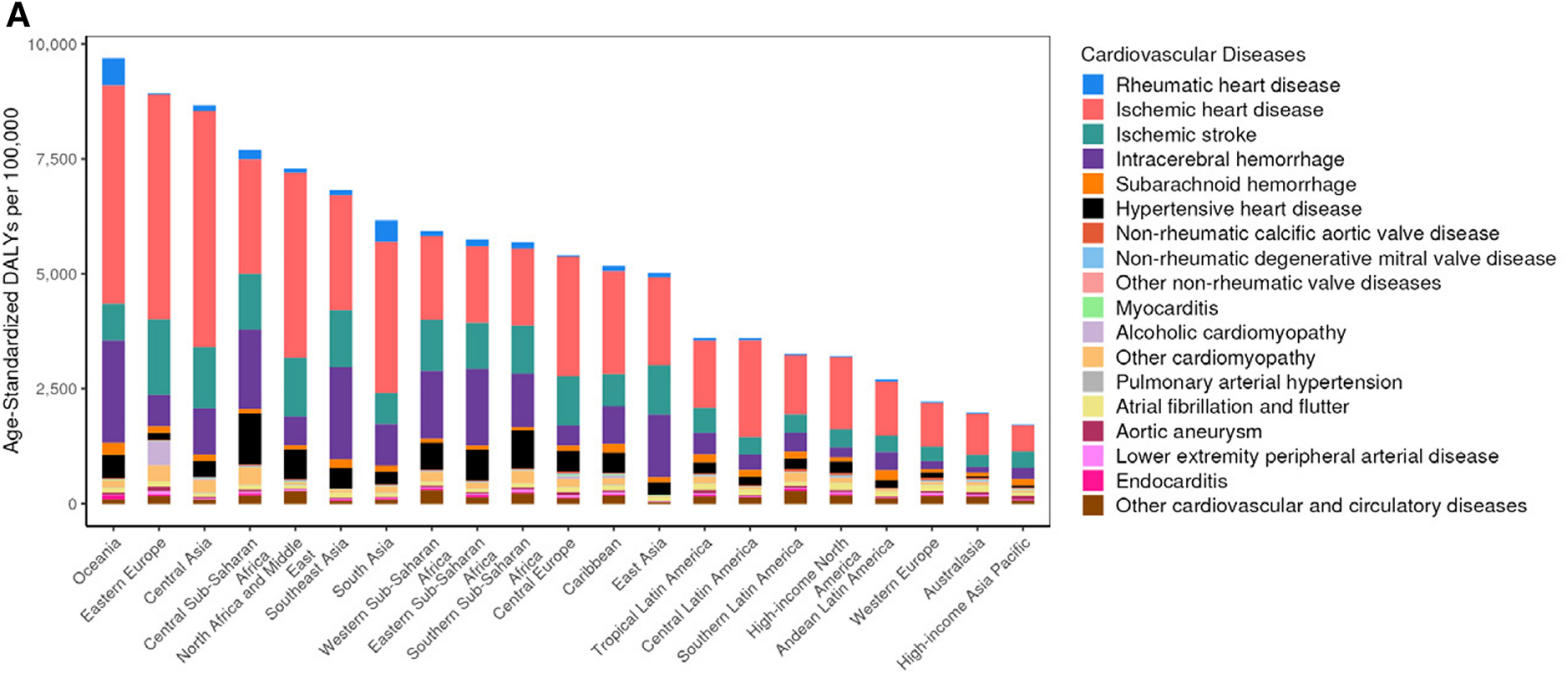
Reprinted from Mensah et al. (2). Copyright (2023), with permission from Elsevier.

Cardiovascular disease (CVD) is a global issue and global deaths from CVD continue to increase (1) (Figure 1). Cardiac Rehabilitation (CR) is a proven interdisciplinary modality based on counseling and physical training aimed to improve exercise capacity and cardiovascular (CV) morbidity and mortality amongst several CVDs (3–5). International guidelines recommend CR with Class I Level of recommendation A/B (based on the diseases) to support its use (6). Most of the leading causes for CVD (Figure 1) would benefit with the intervention of CR (1). However, despite these findings, CR is globally underutilized. This is due to both system and personal barriers, leading patients to not participate or complete a CR program. In addition, there are some groups of patients (i.e., women, elderly patients, and ethnic minorities) in which CR is less prescribed or less used (7). System level barriers include cost, time availability (of both the CR programs and patients), transportation, and personal preference (8). In addition, in the last years, the COVID-19 pandemic made the situation even worse, adding a further barrier in attending or completing CR; indeed, most programs were temporarily closed during the pandemic and several never reopened after the pandemic (9). However, to overcome these limitations, further alternative options to the in-person CR programs have been suggested, with equivalent benefits, safety, and patient satisfaction (10, 11). Alternative options include: Virtual CR (all CR components done via a virtual and on-demand platform), Hybrid (a blend of in-person and virtual), and a home-based CR (12). Compared to traditional CR programs, hybrid models of CR offer several potential advantages. They facilitate eligible patients who are unable to visit rehabilitation centers for various reasons (eliminating the “CR deserts”), decrease medical costs, and improve patient satisfaction and adherence to CR.

In this context, in the present issue of the Frontiers in Cardiovascular Medicine Journal, several manuscripts have been published, dwelling upon various topics related to CR.

In the Western countries, as well as several other industrialized countries, the importance of disease prevention is resulting in a shift in thought, knowledge, and resources. For instance, The Million Hearts Campaign is a national initiative co-led by the Centers for Disease Control and Prevention (CDC) and the Centers for Medicare & Medicaid Services (CMS) taking-action to have at least 70% of eligible patients participate in CR (13, 14). In parallel, in the context of a call for health policymakers to reset the priorities of healthcare resources and provide adequate preventative care, the Chinese government approved the plan named “Healthy China 2030,” which aims to improve national health and prevent disease in China (Zhang et al.). However, despite efforts to increase the availability of CR programs in China, the growth has been slow. In the present issue, Zhang et al. extracted data on 19,896 patients from the online registry platform of the China Society of Cardiopulmonary Prevention and Rehabilitation from February 2012 to December 2021. In this investigation, as in other countries, men and younger patients were more frequently enrolled in CR (15–18). Notably, despite China also having similar system and personal barriers to getting patients referred and enrolled into CR programs, most patients preferred a hospital-based CR. In addition, this investigation pointed out the “CR deserts”, with CR programs being less prominent in the rural areas of China and being offered at tertiary care centers. These issues have resulted in the majority of the Chinese patients diagnosed with CVD being unable to heed the benefits of CR. These disparities will result in a haves and have-not implementation and participation in CR (Zhang et al.).

There are various components of a CR program, which includes exercise, nutrition, psycho-social issues, CV risk factor modification, and education. However, not all of the exercise regimens fit all patients. Therefore, exercise must meld into the individual patient's lifestyle. Efforts to offer various types of exercises must be made, to increase the sustainability and to individualize the exercise regimen. As an example, High intensity interval training (HIIT) is specifically embraced by more elite athletes and women (19) and strength training to rebuild lost skeletal muscle is key for heart failure (HF) patients (20). Furthermore, exercises must be culturally varied in order to embrace our patient's needs.

In the present issue, Zhang et al. performed a meta-analysis of randomized controlled trials aimed to evaluate the effects of Traditional Chinese Exercises (TCE)—such as Tai Chi, Qi Gong, and Ba Duan Jin in patients with myocardial infarction (MI). TCE has become increasingly popular around the world because of their gentle movements, low-risk, easy training, and long-term adherence (Zhang et al.). As a mild muscle- strengthening sport, TCE combines spiritual meditation with moderate postures, musculoskeletal stretching, and deep breathing (21, 22). TCE has been shown in numerous studies to be an effective exercise for CR and to enhance cardiorespiratory health (23, 24). In this investigation based on 21 studies involving 1,890 patients, Zhang et al. showed that the use of TCE was an effective form of exercise in patients after an MI to prevent subsequent CV events and improve patient's emotions and quality of life (QoL) (Zhang et al.).

Heart failure (HF) incidence is increasing (20, 25). HF with reduced ejection fraction (HFrEF) <35% is a qualifying diagnosis that for many is covered by insurance and Medicare. Unfortunately, Medicaid coverage for CR is quite variable from state to state within the United States (US) with very limited CR coverage. Despite heart failure with preserved ejection fraction (HFpEF) increasing, especially in the elderly and women, it is not covered for CR (20, 25). As a set of Tai Chi exercises designed specifically for elderly patients with chronic heart failure, Fu Yang Tai Chi exercises are ideal. Fu Yang Tai Chi stems from the traditions of Tai Chi and incorporates a holistic approach melding the body, mind, and breath, “*emphasizing the importance of spiritual care in moderate exercise, realizing the combination of* *“exercise prescription” and “psychological prescription.”* *The whole set of movements is even and slow, combining movement and stillness, and is a unique connotation of aerobic physical and mental exercise*” (Jiao et al.). However, there are no clinical trials to confirm the effectiveness and safety of the exercises; therefore, Jiao et al. designed a single-center, open label, randomized controlled trial (RCT) to study the effects of Fu Yang Tai Chi as an adjunctive therapy on the QoL of elderly patients with HF. If the results are positive, this therapy could become a good way for older people with HF to exercise at home that is also affordable (Jiao et al.).

In conclusion, the lack of utilization of CR for patients with CVD as a resource to prevent disease is adding insult to injury, salt to the wound. The knowledge is abundant for the benefits of CR to prevent subsequent events in patients with CVD. The COVID pandemic brought to the surface the great need, benefit, efficacy, and safety of alternative options of traditional CR to expand the benefit to the masses of patients with CVD. This global awareness is occurring now. The effects and outcomes will decrease CV events, reduce the barriers to receive CR, and improve CV morbidity and mortality.
